# Effects of Housing System on Dairy Heifer Replacement Cost From Birth to Calving: Evaluating Costs of Confinement, Dry-Lot, and Pasture-Based Systems and Their Impact on Total Rearing Investment

**DOI:** 10.3389/fvets.2020.00625

**Published:** 2020-10-16

**Authors:** Anna Hawkins, K. H. Burdine, D. M. Amaral-Phillips, Joao H. C. Costa

**Affiliations:** ^1^Department of Animal and Food Science, University of Kentucky, Lexington, KY, United States; ^2^Department of Agricultural Economics, University of Kentucky, Lexington, KY, United States

**Keywords:** stochastic model approach, dairy economics, dairy calf, young stock, dairy management, on-farm decision tools

## Abstract

Replacement heifer rearing is critical for the future of dairy operations, to improve genetic merit and maintain herd size. A myriad of options exist on how to manage, feed, and ultimately raise replacement heifers. Pasture is perceived to offer optimal welfare and an economical housing system for replacement animals, but confinement systems are gaining popularity. This study investigates the costs associated with replacement heifer management decisions from birth to calving, considering the factors of housing systems, labor, feed, and health. The objective of this study was to develop an economic model to determine the cost of raising a replacement heifer managed in confinement, dry-lot, and pasture-based scenarios post-weaning. We accounted for variation in feed, labor, and health inputs and quantified the impact of these individual management decisions. An economic simulation with 10,000 iterations were completed for each situation using @Risk and PrecisionTree add-ons (Palisade Corporation, Ithaca, NY) where health incidence, commodity prices, and management variables were made stochastic. Published literature or sample farm data created parameters used in Pert distributions. Costs and biological responses were reflective of published surveys, literature, and market conditions. Management decision inputs had 3 main factors: housing type, ration composition, and labor utilization. Housing systems were calculated separately for confinement, dry-lot, and pasture scenarios. The mean total cost (min, max) to raise a replacement heifer from birth to calving, assuming the same pre-weaning strategy of group housing with an automatic calf feeder, was found to be $1,919.02 ($1,777.25, $2,100.57), $1,593.57 ($1,490.30, $1,737.26), and $1,335.84 ($1,266.69, $1,423.94) for confinement, dry-lot, and pasture, respectively. Total housing cost per replacement heifer was $423.05, $117.96, and $207.96 for confinement, dry-lot, and pasture management systems, respectively. When compared to total cost, housing contributed 21% for confinement, 7% for dry-lot, and 15% for pasture. Upon analysis of all scenarios, utilizing pasture to raise heifers resulted in a lower overall cost when compared to confinement housing options. Percentage breakdowns of feed, labor, housing, and fixed and variable costs provided more information on efficiency rather than total cost, which makes each situation different in relation to on-farm cost. This cost analysis is critical to assisting farms in making decisions in the utilization of their resources for replacement dairy heifers.

## Introduction

Access to pasture is generally assumed to improve welfare for dairy cattle [reviewed by (1)]. However, dairy cattle in many parts of the world are housed on zero grazing or continuous housing systems, especially North America (2, 3). Many of these cows have already entered the milking herd, where confinement housing is used as a tool for more intense management. Pasture is still an important part of the housing system in many of these intensive farms, where pasture is still commonly used in spring and summer to feed heifers and animals with low energy demands, for reasons of lower feed and labor cost.

Replacement heifers are the second largest annual operating expense on the farm, behind only feed cost (4). The cost of raising a replacement heifer is increasing and plays an important role in dairy enterprise economics (5, 6). Heifer raising cost is directly related to feed, housing, and labor demand, which can all contribute to increased cost. In the Netherlands, the difference in actual and perceived cost of heifer retention averaged $898.19 (7). The difference in cost is accounted for in the operation, but it is normally misallocated to another area of dairy expenses. Therefore, determining the true on-farm cost of raising replacement heifers is the first step in better managing these costs.

The decisions that producers make regarding housing options can impact total cost, the development of heifers, and labor utilization. In 2014, the most common housing types for weaned heifers were (1) group housing in a barn and (2) open, dry-lot areas with a barn or shed shelter. While these two housing systems represent over half (54.6%) of all heifers in the United States, over 10 different housing management styles were represented (8). Housing of replacement heifers accounted for 17% of the total cost to raise a weaned heifer in a report from Wisconsin, USA (9). Inputs contributing to housing costs include barn payments, electricity, bedding, and maintenance costs. A potential cost-saving and animal welfare-friendly option would be to raise heifers on pasture. Pasture is utilized by 13.1% of producers for weaned heifers (8). Dairy operations in the Eastern region of the USA are utilizing pasture more than those in the West (10). The adjustment period appears to be quick regardless of whether heifers are kept on pasture for the entire period as a heifer or a select time frame; heifers in the milking herd that were housed previously in confinement for at least a year acclimated to pasture within 3 days.

Analyzing replacement heifer raising costs can uncover additional information about resources utilized on the farm and can assist in evaluating the efficiency of an operation. Feed costs are the primary expense, accounting for 60–73% of all expenses during the rearing period (5, 6). In a 2013 survey of Pennsylvania producers, labor utilization (the second largest contributor to cost) was a clear distinction between efficient and inefficient farms. Farms labeled as efficient were allocating on average $140 in labor resources for each replacement heifer (6). Additionally, biological management decisions can influence the total cost of raising a replacement heifer. For example, raising replacement heifers to be bred to calves at 24 vs. 25 months has the potential to save considerable costs for the dairy enterprise (4). Decreasing cull rates of the milking herd has a direct influence on the cost of the entire heifer raising enterprise, by lowering the required number of heifers to be raised [(4, 11)].

There are multiple options for how to raise replacement heifers on farm, with each decision presenting a unique cost. Many current investment decisions made on dairy operations are based on tradition or intuition, providing an opportunity for more objective methods of investment analysis (12). While there are many factors that influence decisions about dairy heifer raising, including tradition, animal welfare, and environmental concerns, herein we focus on economic efficiency as a primary decision point in heifer raising. The objective of this study was to develop an economic model to determine the cost of raising a replacement heifer managed in confinement, dry-lot, and pasture-based scenarios post-weaning. Furthermore, we account for additional variation in feed, labor, and health inputs and quantify the impact of these individual variables on the total cost.

## Materials and Methods

A heifer cost simulation model was created in Excel 2013 (Microsoft, Redmond, WA, USA) utilizing @RISK add-ons (Palisade Corporation, Ithaca, NY) at the University of Kentucky Dairy Science program. This model serves as the extension to a pre-weaning model described in Hawkins et al. (13). Briefly, the pre-weaning period is an intensive time for raising replacement heifers and total costs for changes ranged from $258.56 to $582.98 (13). Because of the variation in cost during this time period in this analysis, all heifer calves are assumed to follow the growth and cost patterns seen from heifers raised on an automatic calf feeder in group housing, fed milk replacer, and allotted 8 L of milk per day. The total cost found (±SD) was $352.40 ± $16.70 per calf for the pre-weaning period. This accounts for variation in diarrhea and respiratory illness, mortality rate, and weaning age.

**Figure 1:**
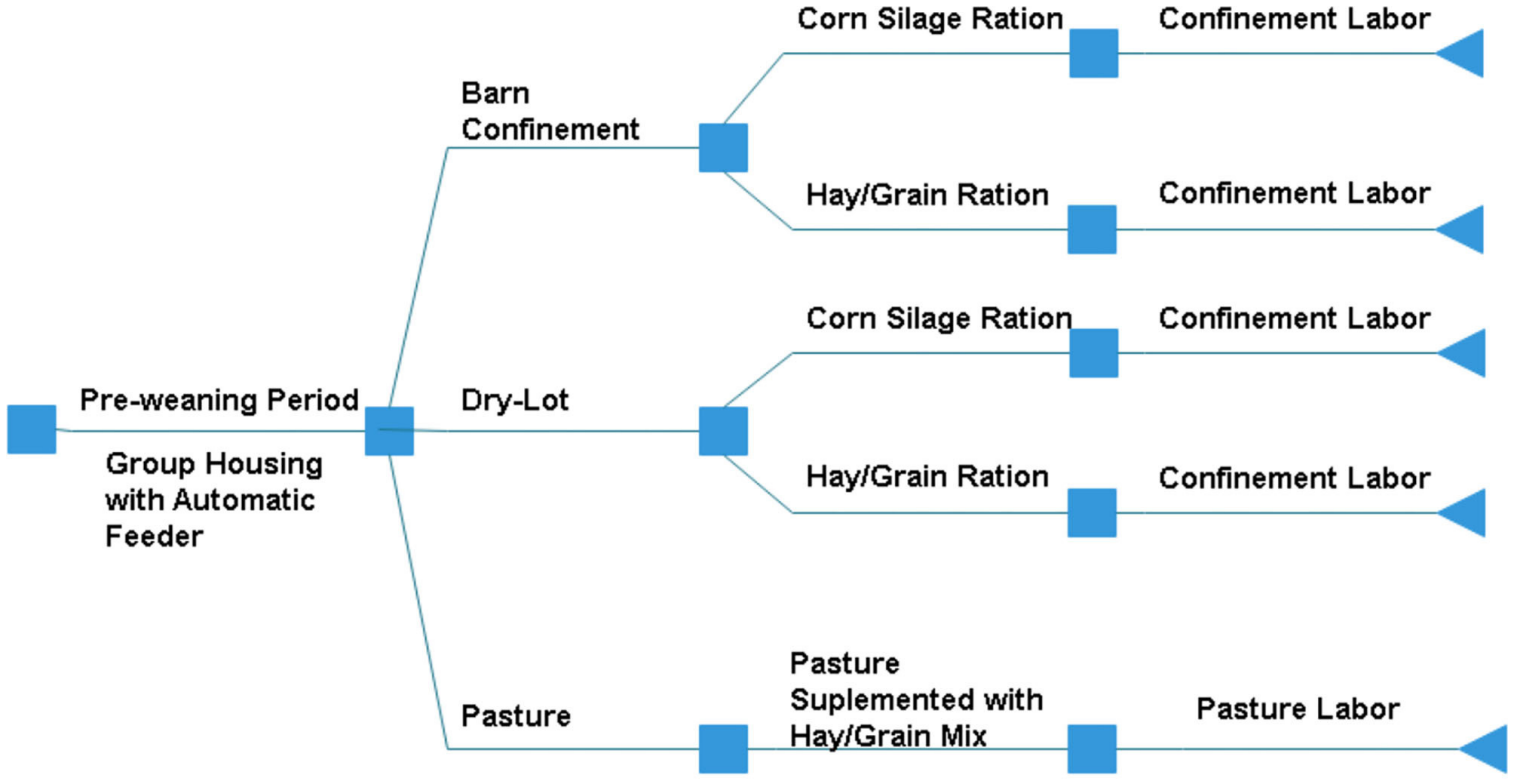
Possible management decision options for producers to raise heifers post-weaning in the model.

Replacement heifer costs were separated into age groups (3–6, 7–10, 11–14, and 15 months to 60 days pre-calving) representing common biological and management changes, such as weaning or reaching puberty, or a change of housing (such as housing heifers on pasture after breeding). Each age group was developed in a new layer within the model to be calculated separately and summed at the end. This opens the possibility of changes within each age category in further model development without changing the rest of the calculations. Management decision options were required for 3 main factors: housing type, ration composition, and labor utilization. The cost associated with each decision was calculated by day; therefore, within each month group, a producer could allocate how many days heifers were utilizing specific resources. This structure allows for more flexibility to account for differences from one farm to the next. Housing could be one of three options: confinement, dry-lot, or pasture. Rations were utilizing corn silage or pasture supplemented with grain. A visual representation of post-weaning management decision pathways for housing, feed, and labor are outlined in Figure 1. Based on previous decisions, only one possible option may be available. For example, if pasture is used within the heifer rearing system, then the only labor option would be time required to care for a heifer on pasture.

Breeding and health-related costs were calculated separately. Health costs per age group were combined with the corresponding month totals, while totals for breeding were incorporated into the final overall cost calculation. All calculated total costs per age group and management style were presented in an overview spreadsheet.

Variables related to health incidence, commodity prices, and on-farm management variables were made stochastic with @RISK simulation. Pert distributions were utilized with parameters set from published literature or sample farm data. A convenience sample of 12 dairy farms located in the states of Ohio and Indiana provided annual financial data to aid in the creation of assumptions. Table 1 outlines the key assumptions made by authors for the calculation of the cost of replacement heifers from weaning to calving.

**Table 1:** Key assumptions presented in the model to determine the cost to raise a replacement dairy heifer from weaning to calving.

**Variable**	**Value**	**Source**
Number of heifers raised annually	1,000	
Hourly employee labor	$14.00	Based on Adcock et al. (14)
Hourly management labor	$22.00	Based on Adcock et al. (14)
Interest rate	7%	
Construction per M^2^ frame	$13.00	(9)
Weaning age	65 days	(9)
Value of newborn calf	$100.00	Based on USDA market reports
Whole milk value (cwt)	$15.00	Based on USDA (8)
Milk replacer value (22.7 kg)	$65.00	Based on average market price
Manure management ($/head/month)	$0.90	(9)
Pasture rental rate (improved pasture)	$40.00	(15)

*Values were found in published literature, extension surveys, and USDA market reports*.

### Housing

Housing costs were calculated separately for three potential options: confinement, dry-lot, and pasture. For the confinement housing scenario, a barn cost per replacement heifer was calculated. The required square meter of barn space was calculated based on the age group and number of animals from the input page. Square meter requirements per replacement heifer began at 2.8 m^2^ at 3–6 months and increased 0.93 m^2^ with each age group (16). The total required m^2^ was multiplied by the construction cost per m^2^ to calculate the barn value. Barn payments were calculated, including interest and depreciation, then broken down by total number of heifers utilizing the barn. Dry-lot and pasture housing scenarios both incorporated land value as the base of housing cost. Pasture, as a housing system, was calculated separately than the nutritional content gained by using pasture as a feedstuff. Average per acre rental rate in Kentucky was used as the assumption to value the land (15). Annual pasture maintained per acre was assumed at $31.50, accounting for seed, equipment, upkeep, and labor. Based on the University of Massachusetts recommendation, 0.5 acres is required per 227 kg of animal and was used to determine the number of replacement heifer per acre. Daily pasture price per animal was calculated using Equation (1). For dry-lot housing, 55.7 m^2^ was required per replacement heifer and used to calculate required spacing. Additionally, dry-lot housing included the calculated investment of 3.71 m^2^ shade per replacement heifer, valued at $0.13 per m^2^ (17). All housing options accounted for water consumption with water valued at $0.75 per cubic meter of water.

(1) $$\begin{array}{l} \left[(\text{Annual Rental Rate per Acre + Annual Pasture Maintenance per Acre}\right)\\ /\text{ 365 days}]\;/\text{ Number of Animals per Acre} \end{array}$$

### Feed

Feed costs were calculated following the nutritional requirement of Holstein dairy heifers in each stage of growth following the NRC (18). Heifer requirements are shown in Table 2. Options for diet formulation included two diet types: R1, comprised of silage, forage, corn, soybean meal, and distillers grain, or R2, which included the utilization of pasture into the diet while supplemented with forage and corn. All rations included a mineral premix and assumed heifers would consume 2.2% of their body weight on dry matter basis. Feed cost was calculated as the average of USDA agriculture commodity market reports from January 2014 to November 2018. Feed cost and rations were both inputs into the model. Therefore, in the available economic model, the user can alter the model to be reflective of their farm or unique conditions.

**Table 2:** Projected weight and nutritional requirements for dairy heifers.

**Age group (months)**	Projected Wt.^*^ (kg)	**DMI (kg/d)**	**ME (Mcal/d)**	**CP %**
3–6	148	4.2	9.6	15.9
7–10	245	6.2	14.1	13.1
11–14	340	7.9	18.2	11.7
15–calving	544	12.2	27.5	13.3

* *Diets were balanced for NRC provided weight requirements which most closely matched projected weights. 150, 250, and 350 kg, respectively*.

The three commodities outlined in Table 2 were made stochastic by assuming a 15% increase or decrease to create a minimum and maximum price. Distribution of the commodity prices is shown in Table 3 for corn, corn silage, and soybeans as a result of the stochastic simulation model. Most values used for feed cost calculations were within 2 standard deviations from the mean. The mean remained the same average value set from USDA published market reports. Shrink of forage and concentrates was accounted for in the daily cost of the feed using Equation (2). An assumption of shrink was made at 10% for silage and forage feedstuff, and 3% for concentrates, based on communications with forage specialists.

(2) $$\begin{array}{l} \text{Total Daily Cost of Individual Feedstuff}/\left(1--\text{\% shrink}\right) \end{array}$$

**Table 3:** The distribution, mean, SD, minimum, and maximum of commodity prices per ton used to calculate feed cost of dairy heifers post-calving.

	**Distribution**	**Mean**	**SD**	**Minimum**	**Maximum**
Corn	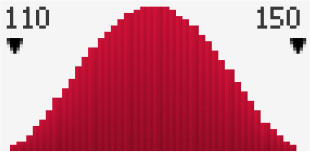	$130.00	$7.37	$111.35	$148.91
Corn Silage	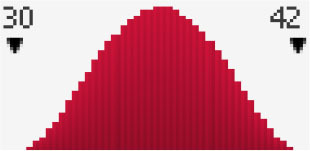	$36.26	$2.06	$30.96	$41.54
Soybean Meal	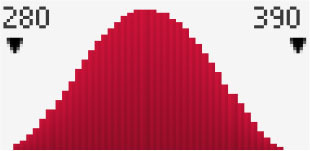	$333.00	$18.88	$284.80	$381.85

*Values were developed using the @RISK. Assumed commodity prices were based on USDA monthly reports from January 2014 to November 2018 for corn and soybeans. Corn silage was valued based on corn commodity price*.

The projected body weight of replacement heifers in each month group was based on a weaning weight of 88 kg and 0.8 kg average daily gain of heifers post-weaning, following results found in Hawkins et al. (13).

### Labor

Required labor hours varied from confinement housing to a pasture-based system. Published surveys of producer-reported time required per heifer were used in the calculation of labor cost. Equation (3) explains how the total labor hours (TLH) were used to determine how many hours of labor are required per replacement heifer.

(3) $$\begin{array}{l} \text{Labor Required per replacement heifer}=TLH\\ /\text{Total Number of days the replacement heifer was in the rearing program} \end{array}$$

To determine the labor cost (LC) within each age group, the total number of days within each month period is multiplied by labor requirement (LR). The resulting variable is the total number of hours of labor required per heifer within each age group (TLR). Equation (4) represents the final step in calculating the cost of labor per heifer (19). Hourly cost associated with more than one employee working on heifers at a time was calculated into the cost.

(4) $$\begin{array}{l} \text{LC}=\text{TLR}{\;}^{*}\text{ Number of Employees}{\;}^{*}\text{ Employee Hourly Wage} \end{array}$$

Pasture-based scenarios followed the same labor calculations outlined above. An assumption was made based on lack of published literature for TLH required per heifer in a pasture-based scenario. 1:02 min was assumed for labor required per heifer; this was broken down from the 3 h of labor requirements per day to care for 175 heifers. The model allows for labor to be provided hours per replacement heifer or total labor hours per day and then divided to get a per replacement heifer cost.

### Health

An external sheet is included in the model to calculate health costs by age group. A standard vaccine protocol was used as the assumed costs. Health related expenses for pre-weaned calves were included in the assumed pre-weaned replacement heifer cost used in all scenarios. Table 4 outlines the vaccines and treatments provided to each age group and subsequently included in the overall cost. Labor requirement for working replacement heifers to provide these injections and treatments through working facilities was accounted for by an additional $0.20 per dose (20). The sum of these expenses resulted in a health cost per age group.

**Table 4:** Outline of the health protocol followed by the authors to create health-related expenses for each age group of heifers.

**Health description**	**Age group (months)**			
	**3–6**	**7–10**	**11–14**	**15–Calving**
Dewormer	X	X	X	X
Fly treatment	X	X	X	X
Respiratory vaccine	X			X
Leptospirosis vaccine	X	X	X	
7-way vaccine	X	X	X	X
*E. coli* vaccine				X
Brucellosis vaccine	X			
*Staphylococcus aureus* vaccine				X
Vitamin A&D				X
Total cost	$11.60	$6.03	$6.37	$8.10

### Breeding

Variation of synch protocols, visual heat detection, or a combination of both was incorporated to account for difference preferences in breeding protocols. After six possible breeding cycles, 7% percent of heifers were assumed to be culled because of unsuccessful breeding. In this situation, Equation (5) was used to determine the additional cost incurred by the remaining heifers on the operation. This accounts for the cost of raising heifers that did not complete the heifer-raising program.

(5) $$\begin{array}{l} {}[\text{Value of Newborn Heifer}+(\text{Total Cost at 13 months}\\ -\text{Springer Heifer Value}{)}^{*}\%\text{Culled}]/\text{Remaining Heifers} \end{array}$$

Heat detection and conception rate were used to determine the number of heifers culled because of breeding performance. In the model, 176 heifers were in the age group to be bred and considered “at risk.” The number inseminated was a function of how many heifers “at risk” were detected to be in heat. The number of pregnant heifers was a result of inseminated heifers multiplied by the conception rate. The difference between “at risk” and pregnant heifers were considered open. This open population would become the “at risk” heifers in the following cycles. Our model allowed for a heifer to complete 6 cycles before she was culled. Services per pregnancy were the sum of all inseminations, divided by the total number of pregnancies. The number of heifers within each group was dependent on how many heifers were culled in the breeding tab.

## Results and Discussion

**Figure 2:**
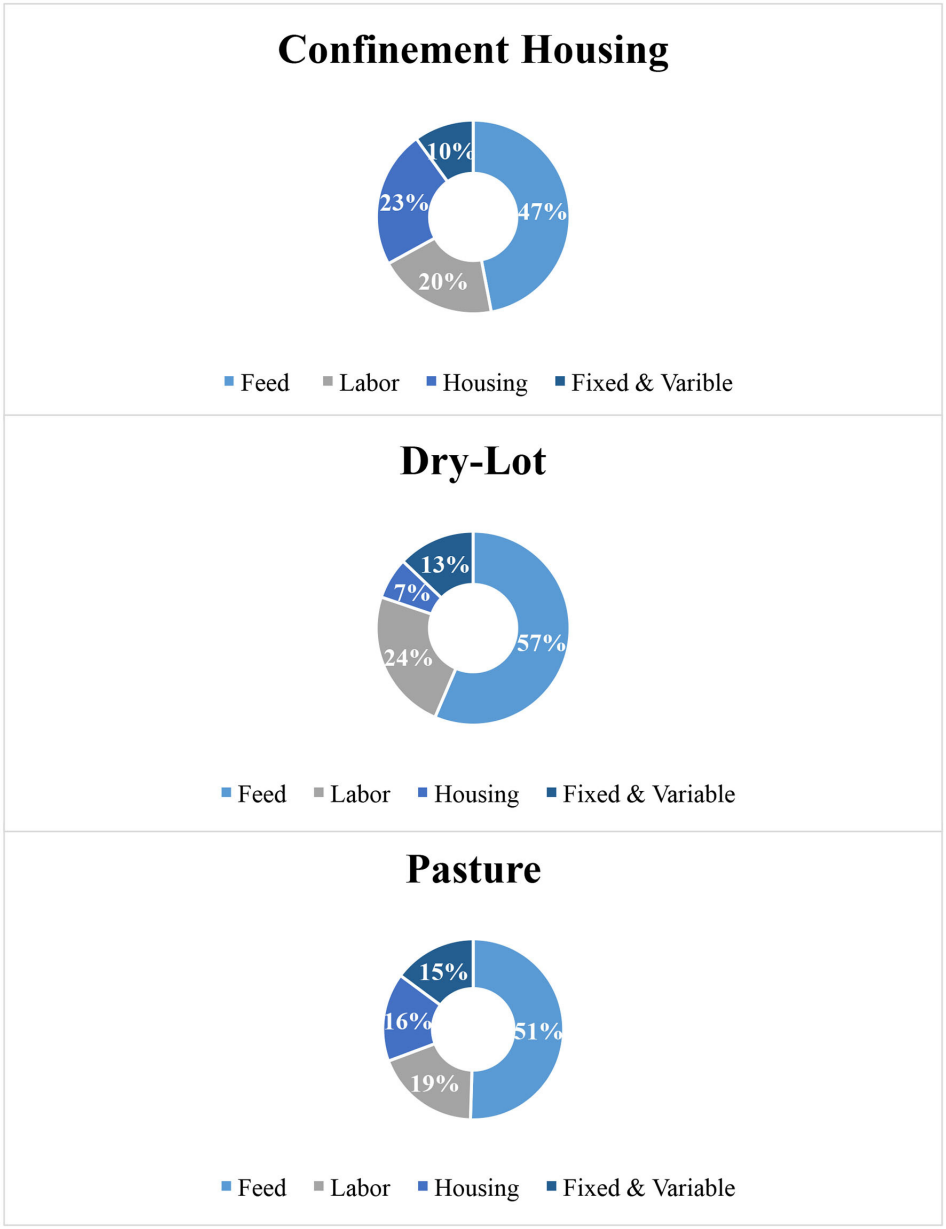
Percentage breakdown of the contribution of housing, feed, labor, and fixed and variable costs in the total replacement heifer rearing period for confinement, dry-lots, and pasture.

The mean total cost (min, max) for a producer to raise a replacement heifer from birth to calving, assuming the same pre-weaning strategy of group housing with an automatic calf feeder, was found to be $1,919.02 ($1,777.25, $2,100.57), $1,593.57 ($1,490.30, $1,737.26), and $1,335.84 ($1,266.69, $1,423.94) for confinement, dry-lots, and pasture management systems, respectively (Table 5). These averages follow the trend of previously published literature, resulting in average values within 1 standard deviation of presented averages (6, 9, 21, 22). The contributions of feed, labor, housing, and fixed and variable costs toward this total cost are reported in Figure 2. The two largest contributing variables to the total cost were feed and labor expenses in all management situations, always representing at least 60% of the total cost.

**Table 5:** Three main housing scenarios were evaluated incorporating the variation represented through stochastic variables.

	**Distribution**	**Mean**	**SD**	**Minimum**	**Maximum**
Confinement	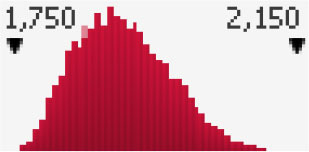	$1,910.02	$58.78	$1,777.25	$2,100.57
Dry-Lot	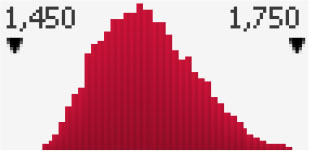	$1,593.57	$44.09	$1,490.30	$1,737.26
Pasture	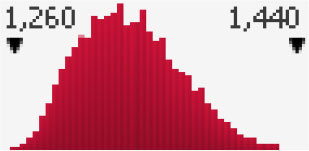	$1,335.84	$28.78	$1,266.69	$1,423.94

*The distribution of total cost, mean, SD, minimum, and maximum is shown for each of the housing types selected*.

### Housing

Total housing cost per replacement heifer was $423.05, $117.96, and $207.96 for confinement, dry-lot, and pasture management systems. When compared to total cost, housing contributed 21% for confinement, 7% for dry-lot, and 15% for pasture. When the sum of variables reported in published surveys is calculated to match the variables presented in our housing group, the average producer-reported housing costs $280. This represented 18% of total allocated cost in an industry-wide report from Wisconsin, USA (9). Most published surveys do not distinguish between housing management system, which may explain the largest cost represented in confinement. Housing cost was the highest for confinement housing because of the additional cost of barn infrastructure. The monthly barn payment per replacement heifer, accounting for interest and depreciation, was $4.81. This model assumes the payment of the barn; therefore, calculated costs may be higher than seen of cash expenditure expenses at the farm. The main contributor for the pasture-based scenario was the value of the land that the replacement heifers were occupying and the associated opportunity cost. With current assumptions, replacement heifers were costing producers $0.06 per day or $1.80 per month for the land as a housing system, excluding additional value of land as a feed source. Because of the nature of dry-lot housing, more heifers could occupy the same acre in comparison to pasture, reducing the land cost per replacement heifer.

### Feed

Feed cost is dependent on input for price per ton and allotment of feed. Total feed cost, under current assumptions, was $932.14, $932.14, and $702.17 for confinement, dry-lot, and pasture management systems, respectively. Confinement and dry-lot scenarios have the same feed cost because both situations are reliant on delivered feed, including a silage ration. As a percentage of the total cost, feed cost contributed 47, 57, and 51% for confinement, dry-lot, and pasture scenarios, respectively. Feed cost is consistently the largest expense on farm in published replacement heifer raising cost, ranging from 51% to over 70% (6, 21). Percentage of feed cost is higher for dry-lots and pastures, partly due to the lower total cost and reduced emphasis on infrastructure found in the housing cost of confinement. This relationship is important when analyzing replacement heifer costs on farm, because we can assume that when comparing percentages of the total cost, confinement will have a lower total percentage of cost in comparison to a pasture setting.

### Labor

Labor was broken down by paid hourly employees and hourly management employees, but labor is reported as the sum of these two expenses. The mean labor expense for confinement, dry-lot, and pasture was $932.14, $932.14, $702.17, respectively. As observed in feed cost, the labor for confinement and dry-lot scenarios are considered the same due to similar time and skill requirements. Labor accounted for 20%, 24%, and 19% of the total cost in confinement, dry-lot, and pasture housing scenarios, respectively. Labor accounted for on average 18.2% of the total cost of Wisconsin dairy producers, just below our calculated percentages (23).

A perceived challenge with this input is determining the time strictly used for caring for replacement heifers. This is particularly important on farms where labor is not hired specifically for the post-weaning replacement heifer period. For example, laborers may split time between feeding and care of replacement heifers and the milking herd, making it difficult to develop a true assumption for the relationship of hourly paid employees and management requirements. We have assumed 10% of the hourly labor was equivalent to the management labor required for replacement heifers. In some situations, management may have varied from this assumption.

### Breeding

Heat detection varied based on management decisions and set reproductive performance. Cost to sync one replacement heifer, utilizing CIDR technology for breeding, was an investment of $19.60 per heifer. Incorporating visual observation into the breeding protocol added an additional cost of $4.68 per replacement heifer. Therefore, heat detection programs utilizing both visual observation and a sync program totaled $24.28 per replacement heifer.

The assumed base reproductive performance was a 65% heat detection rate and a 55% conception rate. Following the herd model of 1,000 heifers annually, 84 replacement heifers would be in the initial “at risk” group of pregnancy. Under our base assumptions after 6 cycles, 7% of the replacement heifers (or 6 heifers) would be culled for reproductive reasons. The cost accrued before breeding for confinement, dry-lot, and pasture management decisions was $1,197.85, $1,063.32, and $927.77, respectively. When distributed over the remaining heifers, there was an additional cost of $8.38, $6.65, $5.13 per replacement heifer for confinement, dry-lot and pasture housing systems, respectively.

Total cost for breeding with a sync protocol and visual heat detection, accounting for additional expenses due to reproductive culls, was $66.95 per replacement heifer. This accounted for 3.4, 4.2, and 5.0% of costs in confinement, dry-lot, and pasture-based management scenarios, respectively. If only visual heat detection was utilized, the percentage of the total cost decreased to 2.2, 2.6, and 3.2% of each management scenario.

### Total Cost

Total replacement heifer raising cost ranged from $1,266 to $2,100 per head. The lowest cost was a result of pasture management decisions, with total cost increasing as infrastructure requirements increased. This model assumed a constant average daily gain across management scenarios, and thus, age at first calving was also consistent. However, many reports of average daily gain of heifers in pasture-based scenarios may be below other housing systems, which could increase the rearing period and increase the presented total costs.

When analyzing replacement heifer cost as an enterprise on the dairy operation on an annual basis, the number of replacement heifers raised can have a large impact on total cost. When the current assumption of the number of replacement heifers raised on farm was reduced by 5% (e.g., 500 heifers annually reduced to 475 replacement heifers), the cost per replacement heifer increased by $85.54, $67.75, $61.89 per heifer for confinement, dry-lot, and pasture management scenarios, respectively. Despite this increase in cost per heifer, the total annual investment in replacement heifers decreased by $7,109, $5,873, and $1,078 annually for each of the respective management scenarios. These results are more variable than the conclusions made by Tozer and Heinrichs (4), who valued a 1% decrease in cull rate of the milking herd which had the potential to decrease overall replacement heifer costs by $1,000–1,500. In addition, our results follow a similar trend found in Mohd Nor et al. (11) where a 5% decrease in cull rate had the potential to decrease replacement heifer costs by $6,500 annually. While heifer raising is often considered a separate enterprise from the dairy herd, management decisions have a large influence on the entire operation.

This study highlights the influences that each factor can have in the different scenarios studied and how it impacts the total rearing cost of replacement dairy heifers. Further studies should investigate the on-farm true cost and the use of economic models for decision-making on-farm.

## Conclusions

Utilizing pasture to raise heifers resulted in a lower overall cost when compared to confinement and dry-lot housing options. Percentage breakdowns of feed, labor, housing, and fixed and variable costs provided more information on efficiency rather than total cost. The model and results presented are dependent on the inputs and assumptions made by the authors. Actual costs calculated may result in higher or lower totals when individual farms utilize the program; nonetheless, the authors determined the model to be highly effective in calculating the cost of raising heifers on an individual farm. This cost analysis is critical to assisting farms in making decisions in the allocation of their resources to raise or purchase replacement dairy heifers. However, a myriad of factors in addition to cost influence decisions around dairy heifer replacement raising on farms, such as tradition, animal welfare, and environmental concerns; these factors in decision-making should be further explored.

## Data Availability Statement

The datasets generated for this study are available on request to the corresponding author.

## Author Contributions

AH and JC: conceptualization and writing—original draft preparation. AH, DA-P, KB, and JC: methodology and writing—review and editing. AH, KB, and JC: formal analysis. AH: investigation. JC: resources and project administration. AH and KB: visualization. KB and JC: supervision. All authors contributed to the article and approved the submitted version.

## Conflict of Interest

The authors declare that the research was conducted in the absence of any commercial or financial relationships that could be construed as a potential conflict of interest.

## References

[1] Charlton GL Rutter SM The behaviour of housed dairy cattle with and without pasture access: a review. Appl Anim Behav Sci. (2017) 192:2–9. 10.1016/j.applanim.2017.05.015

[2] Fulwider WK Grandin T Rollin BE Engle TE Dalsted NL Lamm WD. Survey of dairy management practices on one hundred thirteen North Central and Northeastern United States dairies. J Dairy Sci. (2008) 91:1686–92. 10.3168/jds.2007-0631 18349262

[3] Haskell M Rennie L Bowell V Bell M Lawrence A. Housing system, milk production, and zero-grazing effects on lameness and leg injury in dairy cows. J Dairy Sci. (2006) 89:4259–66. 10.3168/jds.S0022-0302(06)72472-9 17033013

[4] Tozer PR Heinrichs AJ. What affects the costs of raising replacement dairy heifers: a multiple-component analysis. J Dairy Sci. (2001) 84:1836–44. 10.3168/jds.S0022-0302(01)74623-1 11518308

[5] Gabler MT Tozer PR Heinrichs AJ. Development of a cost analysis spreadsheet for calculating the costs to raise a replacement dairy heifer. J Dairy Sci. (2000) 83:1104–9. 10.3168/jds.S0022-0302(00)74975-7 10821586

[6] Heinrichs AJ Jones CM Gray SM Heinrichs PA Cornelisse SA Goodling RC. Identifying efficient dairy heifer producers using production costs and data envelopment analysis. J Dairy Sci. (2013) 96:7355–62. 10.3168/jds.2012-6488 24054291

[7] Mohd Nor N Steeneveld W Derkman THJ Verbruggen MD Evers AG de Haan MHA. The total cost of rearing a heifer on Dutch dairy farms: calculated versus perceived cost. Irish Vet J. (2015) 68:29. 10.1186/s13620-015-0058-x 26675380 PMC4678617

[8] USDA Dairy 2014, Dairy Cattle Management Practices in the United States. Fort Collins, CO: USDA (2016).

[9] Akins MS Cavitt M Hagedorn MA Mills-Lloyd S Kohlman TL Sterry R Economic Costs and Labor Efficiencies Associated with Raising Dairy Calves for Operations Using Individual or Automated Feeding. University of Wisconsin Extension (2017).

[10] Granger L Dairy Heifer Raiser (2011), An Overview of Operations That Specialize in Raising Dairy Heifers. USDA (2012).

[11] Mohd Nor N Steeneveld W Mourits MCM Hogeveen H. The optimal number of heifer calves to be reared as dairy replacements. J Dairy Sci. (2015) 98:861–71. 10.3168/jds.2014-8329 25497803

[12] Bewley JM Boehlje MD Gray AW Hogeveen H Kenyon SJ Eicher SD Stochastic simulation using @Risk for dairy business investment decisions. Agric Finance Rev. (2010) 70:97–125. 10.1108/00021461011042666

[13] Hawkins A Burdine K Amaral-Phillips D Costa JHC. An economic analysis of the costs associated with pre-weaning management strategies for dairy heifers. Animals. (2019) 9:471. 10.3390/ani9070471 31340508 PMC6680651

[14] Adcock F Anderson D Rosson P The Economic Impacts of Immigrant Labor on U.S. Dairy Farms. Center for North American Studies (2015).

[15] Halich G Kindred S Pulliam K Kentucky ANR Agent Land Value and Cash Rent Survey. AEC 2018–90 (2018).

[16] Graves RE Tyson JT McFarland DF Wilson TH Recommendations for Calf and Heifer Housing Dimensions for Holsteins. NRAES-201:278 (2016).

[17] Lardy GP Anderson VL Boyles SL Drylot Beef Cow-Calf Production. North Dakota Agriculture Experient Station, Extenstion Publication AS974 (2017).

[18] National Research Council. Nutrient Requirements of Dairy Cattle: *Seventh Revised Edition*. Washington, DC: The National Academies Press (2001).

[19] Lowe JK Boyer CN Griffith AP Waller JC Bates GE Keyser PD. The cost of feeding bred dairy heifers on native warm-season grasses and harvested feedstuffs. J Dairy Sci. (2016) 99:634–43. 10.3168/jds.2015-9475 26506548

[20] Lima FS De Vries A Risco CA Santos JEP Thatcher WW. Economic comparison of natural service and timed artificial insemination breeding programs in dairy cattle. J Dairy Sci. (2010) 93:4404–13. 10.3168/jds.2009-2789 20723715

[21] Karszes J. C. W. Vokey F Dairy Replacment Programs: Cost & Analysis. Pro-Dairy, Cornell University EB 2008–16 (2008).

[22] Boulton AC Rushton J Wathes DC. An empirical analysis of the cost of rearing dairy heifers from birth to first calving and the time taken to repay these costs. Animal. (2017) 11:1372–80. 10.1017/S1751731117000064 28173887 PMC5523732

[23] Akins MS. Dairy heifer development and nutrition management. Vet Clin N Am Food Anim Pract. (2016) 32:303–17. 10.1016/j.cvfa.2016.01.004 27161393

